# HarvestStat Africa – Harmonized Subnational Crop Statistics for Sub-Saharan Africa

**DOI:** 10.1038/s41597-025-05001-z

**Published:** 2025-04-24

**Authors:** Donghoon Lee, Weston Anderson, Xuan Chen, Frank Davenport, Shraddhanand Shukla, Ritvik Sahajpal, Michael Budde, James Rowland, Jim Verdin, Liangzhi You, Matthieu Ahouangbenon, Kyle Frankel Davis, Endalkachew Kebede, Steffen Ehrmann, Christina Justice, Carsten Meyer

**Affiliations:** 1 Department of Civil Engineering, University of Manitoba, Winnipeg, Manitoba Canada; 2 Climate Hazards Center, Department of Geography, University of California, Santa Barbara, California, USA; 3 Department of Geographical Sciences, University of Maryland, College Park, Maryland USA; 4 NASA Goddard Space Flight Center, Greenbelt, Maryland USA; 5 International Food Policy Research Institute, Washington, DC USA; 6 U.S. Geological Survey, Earth Resources Observation and Science Center, Sioux Falls, SD USA; 7 U.S. Agency for International Development, Washington, DC USA; 8 Department of Geography and Spatial Sciences, University of Delaware, Newark, DE 19716 USA; 9 Department of Plant and Soil Sciences, University of Delaware, Newark, DE 19716 USA; 10 German Centre for Integrative Biodiversity Research (iDiv) Halle-Jena-Leipzig, Leipzig, Germany; 11 Institute of Biology, Leipzig University, Leipzig, Germany; 12 Institute of Geosciences and Geography, Martin Luther University Halle-Wittenberg, Halle (Saale), Germany

**Keywords:** Plant sciences, Agriculture, Environmental sciences, Developing world

## Abstract

Sub-Saharan Africa faces severe agricultural data scarcity amidst high food insecurity and a large agricultural yield gap, making crop production data crucial for understanding and enhancing food systems. To address this gap, HarvestStat Africa presents the largest compilation of open-access subnational crop statistics and time-series across Sub-Saharan Africa. Based on agricultural statistics collated by USAID’s Famine Early Warning Systems Network, the subnational crop statistics are standardized and calibrated across changing administrative units to produce consistent and continuous time-series. The dataset includes 574,204 records, primarily spanning from 1980 to 2022, detailing quantity produced, harvested areas, and yields for 33 countries and 94 crop types, including key cereals in Sub-Saharan Africa such as wheat, maize, rice, sorghum, barley, millet, and fonio. This new dataset enhances our understanding of how climate variability and change influence agricultural production, supports subnational food system analysis, and aids in operational yield forecasting. As an open-source resource, it establishes a precedent for sharing subnational crop statistics to inform decision-making and modeling efforts.

## Background & Summary

Crop production statistics are fundamental to analyzing yield gaps^1,2^, production trends^3,4^, and the effects of climate variability^5–8^, climate extremes^9–11^, and climate change^12–15^ on food systems, as well as knock-on effects of how changes in crop production influence food insecurity and health outcomes. Crop production data is also required to develop operational crop yield monitoring^5,6^ and forecasting systems that support early warning systems^7,8,16–18^.

National-scale crop statistics, such as the data from the Food and Agriculture Organization (FAO) Corporate Statistical Database (FAOSTAT)^19^, span multiple socioeconomic crop production systems and agroecological climate zones. Although these data are an invaluable resource for information on global and regional food production, their coarse spatial resolution limits their utility for spatially detailed climate-crop analyses, crop-yield forecasting, or estimation of yield gaps because they fail to represent spatial variation of yields at the scales where yields respond to climate variability. For this reason, each of the aforementioned studies used either subnational crop yield statistics or national-scale statistics disaggregated to the subnational scale using various downscaling methods and remote sensing^20^. There is broad agreement on the need for increased investment in gathering and managing subnational crop statistics to enhance decisions for food production systems^21^.

Recent international initiatives, such as the FAO-led Global Strategy to Improve Agricultural and Rural Statistics and the “50 by 2030” initiative, have significantly contributed to improving the capacity of national statistics offices to collect timely and accurate agricultural data. In particular, the “50 by 2030” initiative has supported several Sub-Saharan African countries by enhancing their statistical infrastructure and promoting sustainable practices for generating national and subnational crop statistics. However, despite such international support, a substantial funding and technical capacity gap remains, underscoring the need for continued investments to ensure robust and reliable subnational agricultural statistics across the region.

Systematic collation of subnational crop production statistics is particularly important for Sub-Saharan Africa^21^, which contains countries with some of the highest levels of food insecurity and greatest economic dependency on agriculture^22^. In 2022 alone, chronic malnutrition affected nearly 282 million individuals in Sub-Saharan Africa, representing 20% of the region’s population^22^. Sub-Saharan Africa also has the world’s greatest prevalence of agricultural data scarcity due to technical, institutional, and policy barriers^21^, even for key staple crops. The dearth of timely and reliable information on crop production volumes impedes timely responses to food crises and hinders the formulation of public policy. In this context, improved subnational crop production statistics are needed for understanding African food systems, developing crop yield monitoring and forecasting systems, understanding the impacts of climate variability and change, and exploring resilience and adaptation policies to respond to climate change.

In this article, we present HarvestStat Africa, the largest and most comprehensive collection of open-access subnational crop statistics for Sub-Saharan Africa to date. HarvestStat Africa encompasses detailed information on specific crop types, growing seasons, and crop production systems, among other aspects. All crop statistics are harmonized and geolocated to produce consistent and continuous time-series of crop yield, harvested area, and quantity produced. HarvestStat Africa is an open-access, transparent, and standardized compilation of subnational data intended for use in both a research and operational context. The release of the HarvestStat Africa dataset represents the first step in a new generation of community-generated datasets and databases that promote open science through the free and public sharing of subnational crop statistics.

## Methods

Beyond the subnational level of reporting, a key advance of the HarvestStat Africa dataset is the detail provided on the provenance of the data as well as the transparency of subsequent modifications needed to produce continuous time-series of crop production. Providing detailed information on the original source of data and subsequent modifications has been identified as a key barrier to improving the production and use of agricultural data for research and decision making^21^. By collating data in a complex, often data-sparse environment, HarvestStat Africa provides information where it is most needed in a means that is both accessible to end users and suitably flexible for a variety of applications.

**Fig. 1:**
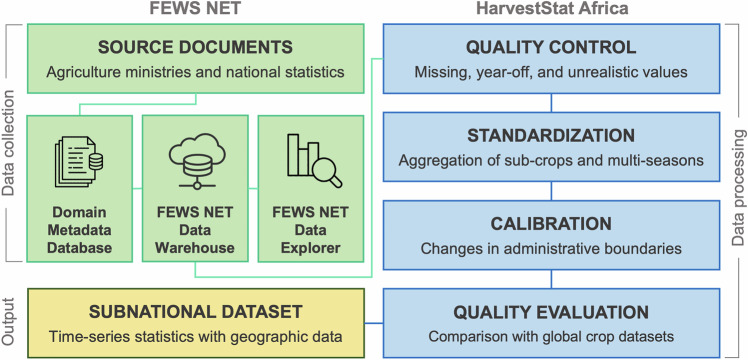
Flowchart illustrates the sequential workflow for data collection, processing, and output within the FEWS NET and HarvestStat Africa frameworks.

The workflow for data collection, processing, and output within HarvestStat Africa is illustrated in Fig. 1, beginning with the USAID’s Famine Early Warning Systems Network (FEWS NET) Data Warehouse (FDW)^23^. Agricultural statistics are first collected by FEWS NET and NASA Harvest, then loaded into the FDW, a centralized hub that facilitates data exploration and visualization via the FEWS NET Data Explorer (FDE)^24^. After the initial data collection phase, the process transitions to the HarvestStat Africa framework, where the data is processed to ensure quality and consistency. This begins with quality control to identify any erroneous or unrealistic values. The data are then standardized into aggregate statistics from various crop types and seasons and calibrated to reflect changes in administrative boundaries. The last step in the HarvestStat Africa process is quality evaluation, where the data are compared with other global crop datasets to ensure consistency and accuracy. The principal output is the subnational dataset, which provides a time-series of crop statistics linked to geographical boundary data.

### A. Data integration and access in FEWS NET

#### Data integration

The FDW^23^ was developed to serve as the central repository for critical data essential to FEWS NET’s efforts in food security and early warning analysis. The data includes statistics related to quantity produced, market prices, exchange rates, and trade. Data in the FDW^23^ can be accessed from the FDE^24^. The FDW^23^ is designed to store subnational quantity produced statistics that are continuously updated from diverse sources, including annual government statistics, reports from agricultural ministries, and tabular data from relevant national agencies. This seamless integration is achieved through monitoring and the maintenance of an extensive database, which includes common metadata and geospatial references.

#### Metadata and data access

Each administrative unit (e.g., state, province, district, etc.) is assigned a unique geocode (FNID) linked to the country’s boundary at a specific point in time. FEWS NET has tracked changes in the names and geometry of administrative boundaries and created a database of historical and current subnational administrative boundaries for a select set of countries, including FEWS-monitored countries (https://fews.net/data/geographic-boundaries, accessed on October 11, 2024). The FDW’s crop statistics also reflect the changes in administrative boundaries in each country.

The metadata within the crop production data domain of the FDW^23^ includes an FNID, a code to identify the crop based on the UN’s Central Product Classification v2 (CPCv2) code^25^, a season name, the season date, information on the crop production system (e.g., irrigated or rainfed), geographic group, and more. After these data undergo internal review (e.g., source reference, tests for plausible accuracy, overlap with existing database) within FEWS NET, they are subsequently uploaded to the FDW^23^. Users are provided with the flexibility to access the data directly from the web platform or through the Application Programming Interface (API). HarvestStat Africa primarily relies on the API for data retrieval, occasionally supplementing it with a small amount of additional data directly from source agencies.

While the FDW^23^ is dedicated to data storage, the FDE^24^ focuses on data access. Within the FDE^24^, data are organized by humanitarian sectors, such as population demographics, market prices, agricultural production, nutrition, and livelihoods, among others, allowing for refined search and filtering capabilities. Additionally, the FDE^24^ provides features for users to explore and validate potentially relevant data through a suite of visualization tools, including tables, graphs, and maps, facilitating the examination of data prior to their export for application.

All crop statistics compiled in HarvestStat Africa are openly available via FDW^23^. The primary source organizations and documents used in HarvestStat Africa are listed in Tables 3, 4, and in Table S1, respectively. The HarvestStat Africa dataset also includes raw crop statistics from FDW^23^, reflecting their state at the time of dataset creation (Table 1).

**Table 1:** Overview of HarvestStat Africa v1.0 dataset^31^ including filenames and descriptions.

Filename	Description
hvstat_africa_data_v1.0.csv	A CSV file containing tabular crop statistics for various countries in Sub-Saharan Africa.
hvstat_africa_boundary_v1.0.gpkg	A GeoPackage file that compiles FEWS NET’s administrative boundaries, aligned with crop statistics via FNID.
README.md	A Markdown file that provides details about the authors, files, and description of data.
fdw_raw_data_v1.0.zip	A zipped file containing raw FDW data.

### B. Data processing in harveststat africa

HarvestStat Africa provides information on yield, area, and quantity produced where available in the FDW^23^ database. However, availability and completeness of the data may vary by country and source document, and given the time required for data collection and processing, the FDW^23^ database may not always immediately reflect the most recent updates. Consequently, countries often exhibit variations in the number of data points related to harvest area, quantity produced, and yield. In such cases, we retain all available data points whenever feasible. Also, some countries report both “planted area” and “harvested area”, and in such instances, we generally report “planted area”. In ten countries we use harvested area due to insufficient planted area data to create a consistent time series. These countries are Uganda, Tanzania, Togo, Niger, Mozambique, Mauritania, Ghana, Ethiopia, Sudan and South Sudan. Data that are unreported or not collected are represented as missing values.

The data processing in HarvestStat Africa primarily focuses on four key processes: quality control, data standardization, calibration of administrative boundaries, and quality evaluation (Fig. 1). We process all countries using the same procedure, with minor revisions tailored to specific issues in each country. For information on quality evaluation, please refer to the Technical Validation section.

#### Quality control of data

During the quality control process, we identify unrealistic and likely misreported values. Although extreme yield shortfalls due to abiotic or biotic stresses are plausible, years with significantly higher yields than the surrounding years are likely outliers. We compute Z-scores for the yield data for each region, crop, and season combination by subtracting the mean and dividing by the standard deviation. We first identify the high-yield tail of each distribution using a threshold of three standard deviations above the mean. We next check whether the high-yielding value is anomalous relative to adjacent values by testing whether it is at least 250% of either the preceding or subsequent value. If a value meets both of these criteria, then we flag it as an outlier in the dataset. We do not apply this criterion unless at least one yield value in the time series is greater than 0.5 tons/ha because such low-yielding systems are expected to have greater variance relative to the mean yield, and identifying outliers in such systems results in erroneous outlier flags.

**Fig. 2:**
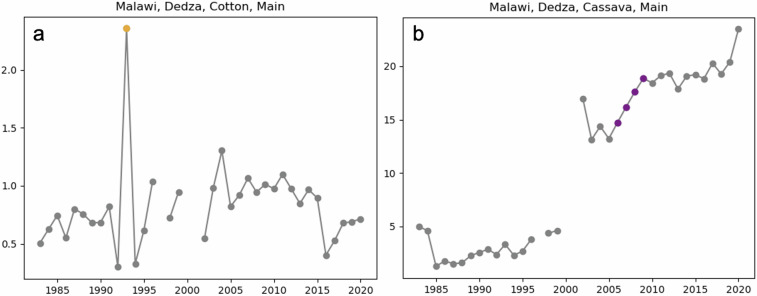
Quality control flags for Dedza, Malawi flagging an outlier value for cotton (**a**) and low variance values for cassava (**b**).

The second type of outlier that we flag are those corresponding to low interannual variance to identify repeated values or values directly following a trend. We identify these values by calculating the second difference of the yield time series. We flag any of the second differences in which three consecutive values were less than 1.5% of the median yield value as being unrealistically low. This is equivalent to identifying at least four values that didn’t meaningfully deviate from the yield trend or four repeated values (Fig. 2). This is the same approach used in Anderson *et al*.^26^ to identify low variance years.

We do not remove the outliers that are identified, but instead clearly identify these values using the “qc_flag” column in the HarvestStat Africa tabular data (refer to Table 2), allowing users to decide how best to process these outliers for their own applications. In addition, we provide our own post-processing analysis of crop statistics through country-specific processing scripts. These scripts are publicly available and accompany the dataset, providing users with the tools to make alternative decisions about data in the post-processing workflow.

**Table 2:** Description of data columns in HarvestStat Africa v1.0 tabular dataset^31^.

Column name	Description
fnid	FEWS NET’s unique geographic unit identifier
country	Name of the country
country_code	ISO 3166-1 alpha-2 country code
admin_1	Name of the first-level administrative unit
admin_2	Name of the second-level administrative unit (if applicable)
product	Name of the crop product
season_name	Name of the growing season
planting_year	Year when planting begins
planting_month	Month when planting begins
harvest_year	Year when harvesting ends
harvest_month	Month when harvesting ends
crop_production_system	Type of crop production system (e.g., irrigated, rainfed, etc.)
qc_flag	Code indicating the result of quality control checks; 0 = no flag, 1 = outlier, 2 = low variance
area	Cropped area (hectares; ha)
production	Crop quantity produced (metric tonnes; mt)
yield	Crop yield (metric tonnes per hectare; mt/ha)

Beyond flagging outliers, we are often unable to judge the accuracy of collected data because the data collected are often the only data available at the subnational level. We do, however, examine the accuracies of HarvestStat Africa and alternative datasets, such as FAOSTAT^19^, to ensure the accuracy of particularly questionable data (see Technical Validation for details). In conjunction with these comparisons, we collaborate closely with FDW^23^ to verify specific metadata.

#### Standardization of data

The FDW^23^ data may include information on crop production systems, population groups, and sub-crops for each crop and country. A sub-crop may refer to different crop varieties or to non-genetic distinctions made on the basis of taste, color, smell, mouth-feel, health benefits, preparation practices, or market preferences. For example, a sub-crop could be a distinction between white and yellow maize or between rice and “broken” rice. For our analysis, we either choose between key sub-crops or aggregate sub-crops as necessary to create a time-series product. In some countries, including Angola, Malawi, and Tanzania, the thematic detail at which certain crop types are reported has changed over time. For example, whereas earlier reports refer to a single category “millet”, this has later been disaggregated into more specific varieties, including “pearl millet” and “finger millet”. To maintain consistency and create a continuous time-series, we have re-aggregated these varieties into the general “millet” category in our dataset. In instances where a sub-crop becomes predominant, less common sub-crops may be omitted. For example, although we report both white and yellow maize in the South Africa data, when combined with all-Africa data, we report only white maize because this is the variety used for human consumption. Depending on data availability, similar decisions are made for the number of seasons and the number of production systems to report. All such decisions are made transparent in our GitHub repository (https://github.com/HarvestStat/HarvestStat-Africa)^27^. Users of the data are free to make a copy of the HarvestStat Africa’s GitHub repository^27^ and make changes to the cleaning and harmonization workflow as they see fit.

In the FDW^23^, the spatial resolution of data changes at times, as in Somalia, Madagascar, Benin, and Tanzania, among other countries. In these cases, producing a continuous time-series often requires aggregation of finer-scale crop statistics to a coarser resolution. In the case of Madagascar, for example, administrative level 3 (district) data from the pre-2012 period were aggregated to administrative level 2 (region) to create a continuous time-series with the post-2012 data. We aggregate the quantity produced and harvested area within the administrative level 2 units and then recalculate yield accordingly. When aggregating data, we only aggregate data when data are available for at least 50% of quantity produced within the coarser resolution administrative unit, which is estimated using a low-frequency Gaussian filter with a kernel standard deviation of three years^26^. We otherwise mark the observation as missing.

Time-series of reported crop statistics may contain changes in spatial and temporal resolution in areas with multiple crop seasons. In Kenya, for example, the FDW^23^ data are reported for a single “annual” season in some years and separately for “short rains” or “long rains” seasons in other years. Here, we maintain this heterogeneity in our product to retain as much fine-resolution data as possible.

#### Spatial calibration

**Fig. 3:**
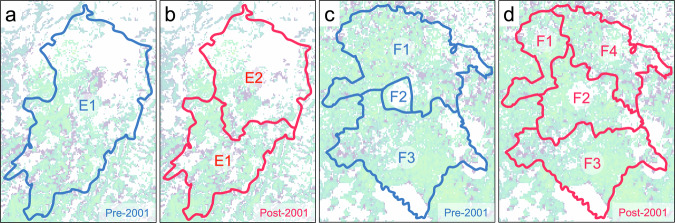
An illustrative example of changes in administrative boundaries in the provinces of Burkina Faso from pre-2001 (left panels; blue lines) to post-2001 (right panels; red lines). The background color represents a crop mask, with green-to-blue colors indicating cropland areas. Top panels (a and b) illustrate Case A, where a single district (E1) splits into two districts (E1 and E2), maintaining equivalent boundary areas. Bottom panels (c and d) illustrate Case B, where three districts (F1, F2, and F3) are reorganized into four districts (F1, F2, F3, and F4), resulting in changes to their boundary areas.

In Sub-Saharan Africa, administrative boundaries have undergone changes over time^28^. These changes within or between countries include splitting, merging, aggregating, and even renaming or changing the administrative levels. Subnational crop statistics often reflect these changes, necessitating the calibration of crop statistics for old administrative units to align with the current administrative units, to ensure their suitability for time-series analysis. We adjust crop statistics (i.e., time-series of quantity produced and harvested area) using the ratio of quantity produced or cropland in each old administrative unit to that of the new administrative units, and then re-calculate crop yield. Two distinct cases are considered:

Case A: This scenario occurs when administrative boundaries change while maintaining their boundary areas. For example, a single district splits into two districts, maintaining equivalent boundary areas (Fig. 3a,b). In such cases, we use the ratios of the mean quantity produced of the new units to calibrate the crop statistics of the old unit, as defined by Eq. (1):

1 $${X}_{i}={X}_{{old}}\left(\frac{{P}_{i}}{\begin{array}{cc}{\sum }_{j}^{n} & {P}_{j}\end{array}}\right)$$

where ${X}_{i}$ is the crop statistic (i.e., time-series of quantity produced and area) in the new administrative unit *i*, ${X}_{{old}}$ is the crop statistics of the old administrative unit, ${P}_{i}$ is the mean crop quantity produced of the new administrative unit *i*, and $$\begin{array}{cc}{\sum }_{j}^{n} & {P}_{j}\end{array}$$ is the sum of quantity produced values in each of the *n* new administrative units. Because these ratios apply uniformly to both quantity produced and harvested area, the re-calculated crop yield remains consistent among the new administrative units. This method is implemented for each crop type to realistically reflect the distinct production characteristics prevalent among various districts.

Case B: This scenario arises when changes in administrative boundaries result in alterations to their respective boundary areas. For instance, an existing district expands to encompass multiple old districts (Fig. 3c,d). Since the ratio of mean quantity produced is not applicable in this case, we use the ratio of cropland area to partially transfer crop productivity from the associated old administrative units to the new administrative unit, as defined by Eq. (2):

2 $${X}_{{new}}={\sum }_{j}^{n}\left({X}_{j}\times \frac{{A}_{{new},j}}{{A}_{j}}\right)$$

where ${A}_{j}$ is the cropland area of the old district *j*, ${A}_{{new},j}$ is the common cropland area between the old and new districts, ${X}_{{new}}$ is the crop statistics of the new administrative unit, and ${X}_{j}$ is the crop statistics of the associated old administrative unit *j*. These ratios are calculated for each of the *n* intersections between the new and the old administrative units. In this case, these ratios are consistently applied to all crop types. The cropland area is extracted from the global cropland map^29^. A similar approach, such as using the arable land class from the land cover map, has been applied to calculate weights for the European subnational crop dataset^30^.

To optimize the calibration process, we focus on significant administrative boundary changes, recognizing that not all changes necessitate calibration. Specifically, we apply calibration when an administrative unit changes its area by at least 10%. Although the calibration is executed automatically, we conduct a visual inspection of all boundary changes in each country. Based on this inspection, we manually modify decisions regarding the type of calibration used, and all such determinations are documented in the country processing scripts. Finally, we compare the total quantities produced and areas before and after calibration to verify the calibration process.

Currently, FEWS NET addresses the ongoing challenge of frequent administrative boundary modifications in Sub-Saharan African countries by systematically identifying these administrative changes and reconstructing historical boundary configurations, linking them directly to crop statistics via the FNID. However, the lack of crop-specific harvested area maps for each year, combined with reliance on a static cropland map, introduces additional uncertainty into the harmonization process. Despite these limitations, our harmonization approach represents a parsimonious and transparent set of assumptions appropriate for the data-scarce environment of Sub-Saharan Africa.

#### Transparency and reproducibility of data processing

Our methods involve minimal corrections to the reported statistics, primarily targeting clear reporting errors or implausible values. All such corrections are documented and openly accessible via the HarvestStat Africa’s GitHub repository^27^, ensuring transparency and enabling reproducibility. The original reported statistics from each country are preserved as closely as possible, with modifications kept to an absolute minimum.

The overall data processing framework is built around open collaboration and transparency. We compile subnational crop statistics directly from the publicly available FDW^23^, process these data within a collaborative GitHub environment, and provide immediate public access to the harmonized, analysis-ready dataset. The FDW^23^ integrates data submitted by multiple partners and actively encourages future data submissions with complete metadata. By openly sharing both the raw and processed datasets, our approach reduces duplication of effort, promotes equitable access to essential agricultural statistics, and provides a robust model that can be scaled and transferred to other regions globally.

## Data Records

The HarvestStat Africa v1.0 dataset is available on Dryad^31^ at 10.5061/dryad.vq83bk42w. The dataset encompasses harmonized crop statistics in tabular format and the administrative boundaries aligned with these statistics, as detailed in Table 1.

The tabular subnational dataset (hvstat_africa_data_v1.0.csv) consists of 16 columns (Table 2), including FEWS NET’s unique geographic unit identifier (FNID), country name, ISO 3166-1 alpha-2 country code, first-level administrative unit name (Admin 1), and second-level administrative unit name (Admin 2, if applicable). Additionally, the dataset records the crop product name, growing season name, and key temporal attributes, including planting year, planting month, harvest year, and harvest month. The planting and harvest timelines are based on FEWS NET’s crop calendars, which are uniform across all administrative units within a country and apply to all crop products. Furthermore, the dataset includes the crop production system type (e.g., irrigated, rainfed), a quality control flag (qc_flag) indicating data validity (0 = no flag, 1 = outlier, 2 = low variance), and crop statistic values for area (hectares; ha), quantity produced (metric tonnes; mt), and yield (metric tonnes per hectare; mt/ha). The dataset is linked to administrative boundary data (hvstat_africa_boundary_v1.0.gpkg) through FNID, ensuring alignment between statistical records and geographic regions. The administrative boundaries are synthesized from individual country boundary files, allowing for seamless spatial integration with crop statistics.

**Fig. 4:**
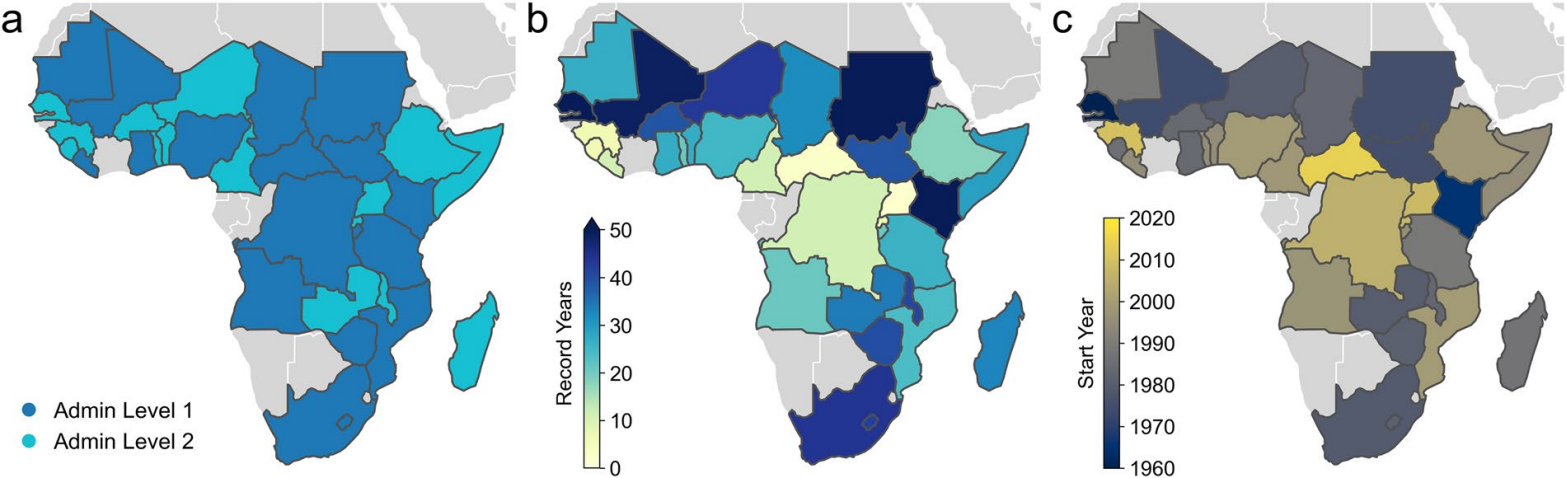
(**a**) Administrative levels, (**b**) number of recorded years, and (**c**) first year covered by processed crop statistics in HarvestStat Africa v1.0^31^. The data for (**b,c**) encompass all available crop types.

Figure 4 and Tables 3, 4 provide details on the countries whose data are processed (refer to Table S2 for additional details on the number of years recorded for each crop). The HarvesStat Africa v1.0^31^ encompasses a total of 574,204 records, comprising 198,346 records for quantity produced, 190,428 for area, and 185,430 for yield. In total, 33 countries have been included, comprising 18 with data at administrative level 1 and 15 at administrative level 2 (Fig. 4). Spatial calibration has been implemented in 19 countries. Although administrative boundaries in these countries typically underwent 1–2 changes, some countries, like Ethiopia, have required up to 6 boundary calibrations over a span of 25 years. HarvestStat Africa v1.0^31^ includes data on 94 crop types. Although several crop types belong to the same crop class, we retain the specific crop types as reported in the source document (e.g., Cotton (American) and Cotton (Egyptian)). Data on multiple growing seasons and multiple crop production systems are reported in 22 and 11 countries, respectively.

**Table 3:** Overview of countries and processed subnational crop data in HarvestStat Africa v1.0^31^.

Country	Administrative level (Local name)	Spatial calibration	# of seasons	# of crops	# of CPS	Primary source organization(s)
Angola	1 (Province)	No	1	26	1	Ministry of Agriculture and Forestry, Angola
Benin	2 (Commune)	Yes	2	30	1	Ministere de l’Agriculture, Direction de la Statistique Agricole, Benin
Burkina Faso	2 (Province)	Yes	2	15	4	Ministère de l’Agriculture, des Ressources animales et halieutiques, Burkina Faso
Burundi	1 (Province)	Yes	3	20	1	Institut de Statistiques et d’Etudes Economiques du Burundi
Central African Republic (CAF)	1 (Prefecture)	No	1	5	1	Food and Agriculture Organization/World Food Programme, Central African Republic
Cameroon	2 (Division)	No	5	23	1	Ministere de l’agriculture, Cameroun
Chad	1 (Region)	Yes	2	13	1	Ministry of Agriculture and Irrigation, Chad
Democratic Republic of the Congo (DRC)	1 (Province)	Yes	1	5	1	Ministère de l’agriculture pêche et élevage, Democratic Republic of the Congo
Ethiopia	2 (Zone)	Yes	1	45	1	Ministry of Agriculture, Ethiopia
Ghana	1 (Region)	Yes	2	12	1	Ministry of Food and Agriculture, Ghana
Guinea	2 (Prefecture)	No	1	4	1	L’Agence Nationale des Statistiques Agricoles et Alimentaires, Guinea
Kenya	1 (County)	Yes	3	39	1	Ministry of Agricultural and Livestock Development, Kenya
Lesotho	1 (District)	No	2	6	2	Lesotho Bureau of Statistics, Lesotho
Liberia	1 (County)	Yes	1	2	1	Ministry of Agriculture, Liberia
Madagascar	2 (Region)	Yes	1	37	1	Ministry of Agriculture, Madagascar
Malawi	2 (District)	Yes	3	29	3	Ministry of Agriculture, Irrigation and Water Development, Malawi
Mali	1 (Region)	Yes	1	18	1	Ministere De L’agriculture, Mali

The “CPS” stands for crop production systems.

**Table 4:** Continued from Table 3.

Country	Administrative level (Local name)	Spatial calibration	# of seasons	# of crops	# of CPS	Primary source organization(s)
Mauritania	1 (Region)	No	8	7	6	Ministry of Rural Development, Mauritania
Mozambique	1 (Province)	No	4	31	3	Ministério da Agricultura e Segurança Alimentar, Mozambique
Niger	2 (Department)	Yes	2	36	3	Ministere de l’Agriculture, Niger
Nigeria	1 (State)	No	2	20	1	National Agricultural Extension and Research Liaison Services, Nigeria
Rwanda	2 (District)	No	3	30	1	Ministry of Agriculture and Animal Resources, Rwanda
Senegal	2 (Department)	Yes	2	10	3	Agence Nationale de la Statistique et de la Demographie, Senegal
Sierra Leone	2 (District)	No	1	12	1	Ministry of Agriculture, Forestry and Food Security, Sierra Leone
Somalia	2 (District)	No	4	10	3	Food Security and Nutrition Analysis Unit, Somalia
South Africa	1 (Province)	No	2	10	1	Crop Estimates Committee, Department of Agriculture, Forest and Fisheries, South Africa
South Sudan	1 (State)	Yes	2	8	4	Food and Agriculture Organization/World Food Programme, Government of South Sudan
Sudan	1 (State)	Yes	2	10	7	Federal Ministry of Agriculture and Forestry, Sudan
Tanzania	1 (Region)	Yes	4	25	1	Ministry of Agriculture, Food Security and Cooperatives, Tanzania
Togo	2 (Prefecture)	Yes	2	12	1	Direction des Statistiques Agricoles, de l’Informatique et de la Documentation, Togo
Uganda	2 (District)	No	3	15	1	Ministry of Agriculture, Animal Industry and Fisheries, Uganda
Zambia	2 (District)	Yes	1	19	1	Ministry of Agriculture and The Central Statistics Office, Zambia
Zimbabwe	1 (Province)	No	1	14	8	Food and Agriculture Organization/World Food Programme, Ministry of Lands, Agriculture, Fisheries, Water and Rural Development, Zimbabwe

**Fig. 5:**
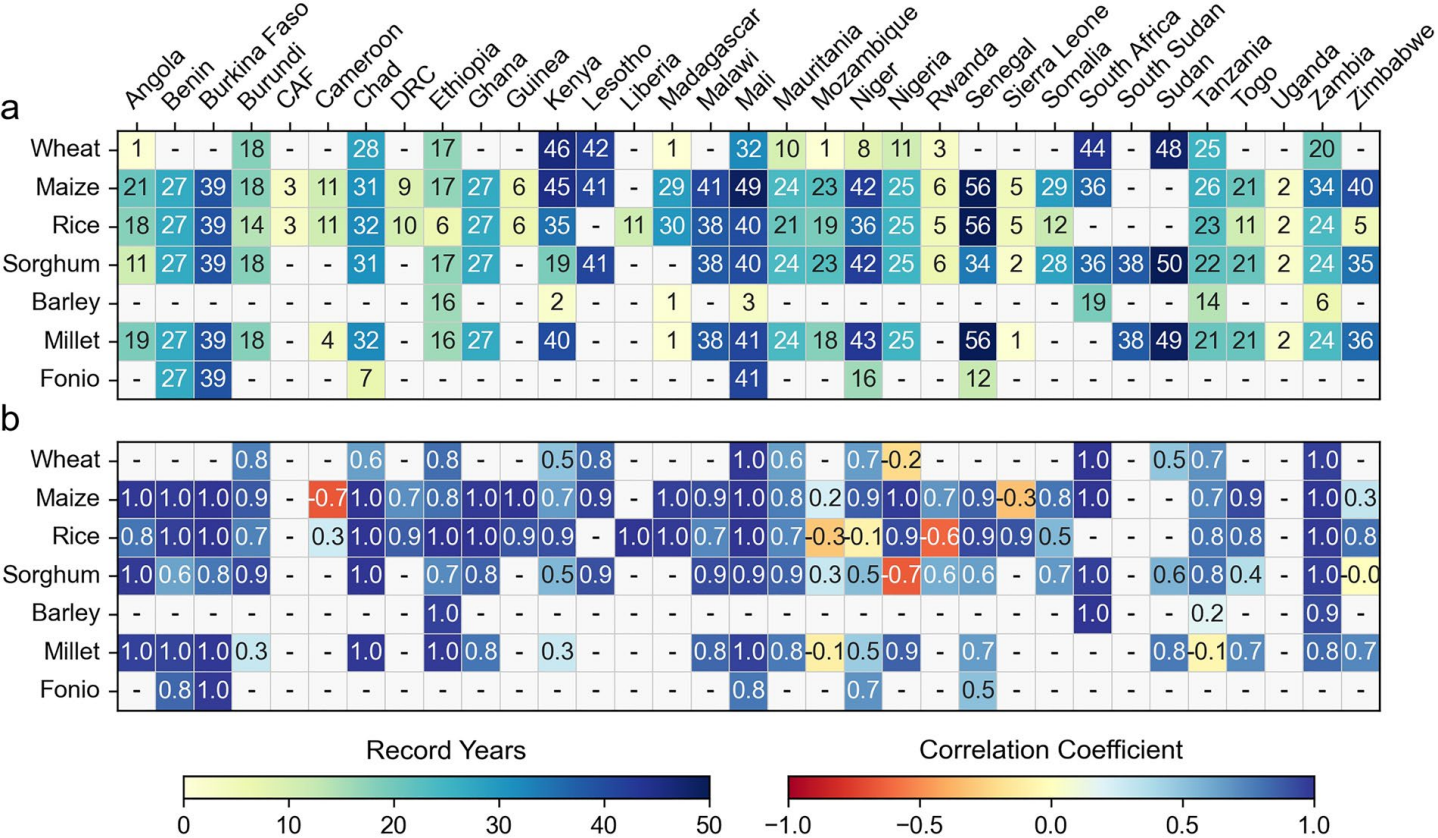
(**a**) Number of years with data for records of quantity produced and (**b**) correlation coefficient of quantity produced at the national scale crop productions between the HarvestStat Africa v1.0^31^ and FAOSTAT dataset^19^ for seven grain types. The record years do not necessarily represent consecutive years. The correlation was calculated when at least 5 years of data were available.

Figure 5a depicts the number of recorded years with data for quantity produced for seven grain types. On average, grain crops, such as wheat, maize, rice, sorghum, barley, millet, and fonio, demonstrate a more extensive record presence, with 23 years of records across all countries, highlighting their significant role in diverse agricultural assessments. In contrast, vegetables and fruits exhibit the lowest average record span, ranging from 6 to 9 years. Other crop groups show varying numbers of years of reliable records: oilseeds and oleaginous fruits (18 years), edible roots and tubers (13 years), pulses (17 years), and sugar crops (14 years). While certain countries, including Burkina Faso, Burundi, Cameroon, Ethiopia, Madagascar, Malawi, Mali, Niger, and Nigeria, have comprehensive records spanning most crop types, countries such as the Central African Republic, Guinea, and Uganda present limited recorded years.

As a dynamic dataset, HarvestStat Africa will be further curated to ensure it remains up-to-date and reliable. These updates will include additions of new data and revisions of existing data from FDW^23^, as well as further data corrections and improvements within the FDW/HarvestStat Africa framework. To facilitate transparency and user access to these modifications, both the country-specific scripts and the updated output dataset will be maintained in the HarvestStat Africa’s GitHub repository^27^. This approach ensures that users can easily track and identify any changes between versions and enhances the dataset’s utility and reliability.

## Technical Validation

### Evaluation approach for plausibility

In this section, we describe how we assessed the data quality, consistency, and unique advantages of HarvestStat Africa by comparing its outputs with other comparable global datasets. For tabular data in HarvestStat Africa, we correlate the national quantity produced figures with national statistics from FAOSTAT^19^. Although HarvestStat Africa’s source documents are considered direct observations, verifying the consistency of HarvestStat Africa with FAOSTAT is essential to identify and rectify any potential discrepancies. Moreover, we conduct a spatial analysis of HarvestStat Africa data by comparing them with Earthstat^32^, Global Data of Historical Yields (GDHY)^33^, and the International Food Policy Research Institute’s Spatial Production Allocation Model (SPAM)^34^. This analysis highlights the ability of HarvestStat Africa to represent the reported spatial patterns of crop yield and its trends on a subnational scale, which is different from national-scale approaches typically used in other datasets^32–34^.

### Comparison to FAOSTAT

Figure 5b shows Pearson correlations of national annual quantity produced time-series between HarvestStat Africa v1.0^31^ and FAOSTAT^19^, with HarvestStat Africa data entries spanning less than five years being omitted for clarity. Additionally, correlation is not calculated in cases where FAOSTAT lacks data (e.g., Fonio in Chad). In instances of multiple growing seasons and crop production systems, as identified for countries like Burundi, Kenya, and Somalia (see Tables 3, 4), seasonal quantities produced are aggregated into annual figures for direct comparison with annual quantity produced data from FAOSTAT^19^. Spatial calibration and standardization processes for HarvestStat Africa do not influence the comparison of national annual quantity produced figures. The analysis predominantly reveals positive correlations, with a median correlation coefficient of 0.77 for all crops, indicating a high level of consistency between HarvestStat Africa v1.0^31^ and FAOSTAT^19^. Specifically, grain crops exhibit a median correlation coefficient of 0.82, indicating substantial agreement. Notably, primary staple crops in each country demonstrate strong correlations (ranging from 0.9 to 1.0). Several countries, including Burkina Faso, Lesotho, Malawi, Chad, South Africa, and Zambia, show high levels of agreement with FAOSTAT^19^ across most crop categories, with correlation coefficients exceeding 0.8 (Fig. 3b).

In contrast, non-grain crops exhibit a wider range of correlation levels with FAOSTAT, ranging from −0.8 to 1.0. The source of these variations is difficult to identify without an independent dataset, but variations may arise from data quality issues with either the subnational data in HarvestStat Africa or FAOSTAT. Direct comparisons may be challenging for certain crops, given FAOSTAT’s aggregation of multiple crops within a single category (e.g., carrots/turnips and onions/shallots), and instances where HarvestStat Africa categorizes crops more granularly or broadly than FAOSTAT. Despite FAOSTAT being regarded as the foremost global dataset for crop production data, approximately 30% of its entries are flagged as estimated, imputed, or unofficial figures. Hence, discrepancies do not always imply inaccuracies in HarvestStat Africa data. Overall, the predominantly high positive correlations underscore the consistency and reliability of agricultural data across a broad spectrum of crops and countries within the HarvestStat Africa framework, as benchmarked against FAOSTAT.

### Comparison to gridded data products on yield datasets

**Fig. 6:**
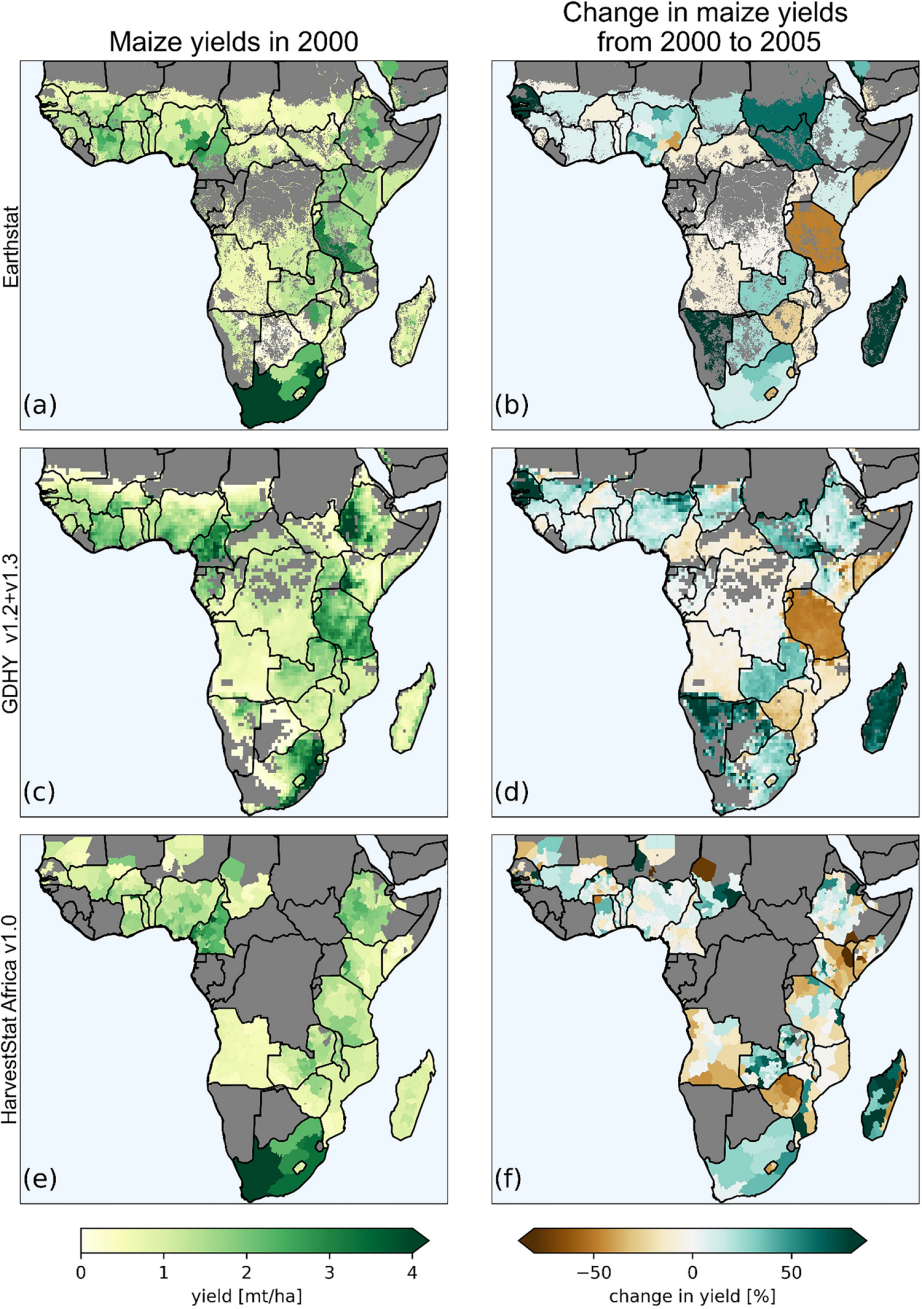
Comparison of (**a**) the EarthStat dataset^32^, (**c**) GDHY v1.3 dataset^33^, and (**e**) HarvestStat Africa v1.0 dataset^31^ for maize yields around the year 2000 (1998–2002) (**a,c,e**) and in the change of maize yields from 2000 (1998–2002) to 2005 (2003–2007) for each dataset (**b,d,f**).

HarvestStat Africa is not the only publicly available subnational crop yield dataset but, at the time of publication, is the only dataset that exclusively comprises subnational data in the African domain, providing a higher resolution in both time and space. To understand how the HarvestStat Africa v1.0^31^ compares to other datasets, we compare HarvestStat Africa v1.0 maize yields around the year 2000 to Earthstat^32^ and GDHY v1.3^33^ (Fig. 6a,c,e).

Each of the aforementioned subnational datasets uses a different approach to produce subnational crop yield estimates. The GDHY v1.3 dataset^33^ begins with FAO country-level statistics before disaggregating crop yields to the pixel-level using the fraction of photosynthetically available radiation (fPAR) and leaf area index (LAI) during the growing season as an indication of subnational vegetative health^33^. The EarthStat dataset^32^ also blends FAO country-level data with subnational data by using FAO data to fill missing gaps in the collected subnational statistics and by scaling the nearest five years of available subnational data to FAO estimates at the country level. Portions of the data used in the EarthStat dataset^32^ are available from the EarthStat website (http://www.earthstat.org; accessed on Mar 11, 2025). A final product that we do not compare against is the SPAM dataset^34^, which combines subnational crop statistics with information on cropland extent, climate, and socioeconomic development to produce distributions of crop yields, harvested areas, and production at a pixel scale^34^. We do not compare against the SPAM dataset^34^ because it is not designed to be used in a time-series analysis.

Each of the existing subnational crop yield datasets produces data that have an apparent subnational resolution. However, due to the reliance on country-level data for gap-filling and scaling, the true resolution of the existing products remains unclear. The EarthStat dataset^32^, for example, does not specify where the product uses subnational data vs gap-filling with country-level data. This raises the possibility that it is, in fact, primarily a national-scale product in some places. Figure 6 illustrates the effective resolution of the data using the change in yields from around the year 2000 (1998–2002) to that around the year 2005 (2003–2007). Uniform yield differences across many countries–which are derived from the use of country-level FAOSTAT data–dominate the interannual variability of both EarthStat^32^ and GDHY^33^ (Fig. 6b,d). Because the GDHY dataset^33^ starts with the time-series of country-level FAOSTAT yields, the spatial variability follows the vegetative health indices while the interannual variability of the data is dominated by the underlying country-level FAOSTAT data. The authors clearly acknowledge this point, stating that “the spatial variation in modelled yields in a country followed that in [net primary productivity], whereas the temporal variation in modelled yields basically followed those in the FAO data”^3^. In the EarthStat dataset^32^, the country-level temporal resolution is likely a result of subnational data scarcity in Africa in the dataset, which would necessitate gap-filling missing years with pattern-scaled FAO data. Both the EarthStat dataset^32^ and GDHY dataset^33^ demonstrate temporal subnational resolution in some locations, meaning that while there are different yield levels in different administrative units (Fig. 6a,c,e), all administrative units within a country experience the same yield changes from year-to-year (Fig. 6b,d,f). The EarthStat dataset^32^ shows subnational temporal resolutions over Nigeria, for example, and GDHY dataset^33^ well differentiates yield levels that vary across Kenya as is present also in the subnational data of HarvestStat Africa. Subnational HarvestStat Africa data are presented without in-filling of years and areas where subnational data are unavailable to allow for the most appropriate downstream use of the data in, e.g., panel regression models^35^.

### Limitations and uncertainties of crop statistics

Subnational crop statistics in Sub-Saharan Africa may exhibit inherent uncertainty due to technical errors, such as sampling, processing, and coverage errors in agricultural census statistics^21,36^. While certain source documents explain their sampling methods for crop production reporting, others lack such information entirely. Measuring harvested area accurately is challenging without advanced techniques^37^, which are often not available in various regions, especially in past decades^36^. It is common for one indicator, such as harvested area, to be inferred from the other indicators.

The availability of crop statistics in Sub-Saharan Africa is often discontinuous in both space and time. Data may not be collected in every administrative unit every year and subnational estimates are often not available for every year. The limited resources available for data collection of crop production and yield in some countries may also affect data quantity and quality in subnational statistics. This may manifest in data being estimated based on sparse samples taken from, e.g., farmer estimates or crop cut methods, or in limited or infrequent collection of subnational data. An additional systematic bias in some countries is that during particular years (e.g., poor crop-growing conditions) surveyors are not sent to areas of crop failure to save time and money on petrol, resulting in a value of “not collected” rather than a zero or near-zero quantity produced value. Additionally, figures from previous years are sometimes used to replace unobserved statistics. An example of this is the 2021/2022 statistics for the Tigray region in Ethiopia, which was impacted by the Tigray conflict starting in 2020^38^.

### Future validation opportunities

While HarvestStat Africa focuses on harmonizing and openly disseminating subnational agricultural statistics, future validation could benefit from comparisons with independent sources, such as field measurement and remotely sensed indicators like the Normalized Difference Vegetation Index (NDVI). Previous studies have demonstrated the potential for satellite-derived metrics to independently assess crop yields in Sub-Saharan Africa^39,40^. However, comparing remotely sensed indices and reported crop statistics involves methodological complexities arising from variations in cropping systems, crop phenology, and farmer-reporting accuracy^41^. Nonetheless, incorporating these independent indicators could validate the accuracy of subnational statistics and highlight discrepancies potentially resulting from reporting biases or methodological inconsistencies. Such validation efforts could ultimately enhance the reliability of agricultural data across the region.

## Supplementary information

Supplementary Information for “HarvestStat Africa – Harmonized Subnational Crop Statistics for Sub-Saharan Africa”

## Data Availability

Our custom code is available in the HarvestStat Africa’s GitHub repository^27^. It comprises data preparation, individual country processing scripts, and an aggregation process for consolidating output files. This setup ensures transparent and replicable data handling from retrieval to final output generation.

## References

[1] Neumann, K., Verburg, P. H., Stehfest, E. & Müller, C. The yield gap of global grain production: A spatial analysis. *Agric. Syst.* **103**, 316–326 (2010).

[2] Van Ittersum, M. K. *et al*. Yield gap analysis with local to global relevance—A review. *Field Crops Res.* **143**, 4–17 (2013).

[3] Iizumi, T. *et al*. Historical changes in global yields: major cereal and legume crops from 1982 to 2006. *Glob. Ecol. Biogeogr.* **23**, 346–357 (2014).

[4] Ray, D. K., Mueller, N. D., West, P. C. & Foley, J. A. Yield Trends Are Insufficient to Double Global Crop Production by 2050. *PLoS ONE* **8**, e66428 (2013). 23840465 10.1371/journal.pone.0066428 PMC3686737

[5] Becker-Reshef, I. *et al*. Prior Season Crop Type Masks for Winter Wheat Yield Forecasting: A US Case Study. *Remote Sens.* **10**, 1659 (2018).

[6] Becker-Reshef, I. *et al*. Monitoring Global Croplands with Coarse Resolution Earth Observations: The Global Agriculture Monitoring (GLAM) Project. *Remote Sens.* **2**, 1589–1609 (2010).

[7] Fritz, S. *et al*. A comparison of global agricultural monitoring systems and current gaps. *Agric. Syst.* **168**, 258–272 (2019).

[8] Funk, C. *et al*. Recognizing the Famine Early Warning Systems Network: Over 30 Years of Drought Early Warning Science Advances and Partnerships Promoting Global Food Security. *Bull. Am. Meteorol. Soc.* **100**, 1011–1027 (2019).

[9] Lesk, C., Coffel, E. & Horton, R. Net benefits to US soy and maize yields from intensifying hourly rainfall. *Nat. Clim. Change* **10**, 819–822 (2020).

[10] Ray, D. K., Gerber, J. S., MacDonald, G. K. & West, P. C. Climate variation explains a third of global crop yield variability. *Nat. Commun.* **6**, 5989 (2015). 25609225 10.1038/ncomms6989 PMC4354156

[11] Vogel, E. *et al*. The effects of climate extremes on global agricultural yields. *Environ. Res. Lett.* **14**, 054010 (2019).

[12] Iizumi, T. *et al*. Crop production losses associated with anthropogenic climate change for 1981–2010 compared with preindustrial levels. *Int. J. Climatol.* **38**, 5405–5417 (2018).

[13] Lesk, C. *et al*. Compound heat and moisture extreme impacts on global crop yields under climate change. *Nat. Rev. Earth Environ.* **3**, 872–889 (2022).

[14] Ray, D. K. *et al*. Climate change has likely already affected global food production. *PLOS ONE* **14**, e0217148 (2019). 31150427 10.1371/journal.pone.0217148 PMC6544233

[15] Tigchelaar, M., Battisti, D. S., Naylor, R. L. & Ray, D. K. Future warming increases probability of globally synchronized maize production shocks. *Proc. Natl. Acad. Sci.* **115**, 6644–6649 (2018). 29891651 10.1073/pnas.1718031115 PMC6042138

[16] Nakalembe, C. *et al*. A review of satellite-based global agricultural monitoring systems available for Africa. *Glob. Food Secur.* **29**, 100543 (2021).

[17] Lee, D. *et al*. Maize yield forecasts for Sub-Saharan Africa using Earth Observation data and machine learning. *Glob. Food Secur.* **33**, 100643 (2022).

[18] Davenport, F. M. *et al*. Using out-of-sample yield forecast experiments to evaluate which earth observation products best indicate end of season maize yields. *Environ. Res. Lett.* **14**, 124095 (2019).

[19] FAO. FAOSTAT. https://www.fao.org/faostat/en/#data. (2023).

[20] Szyniszewska, A. M. CassavaMap, a fine-resolution disaggregation of cassava production and harvested area in Africa in 2014. *Sci. Data* **7**, 159 (2020). 32461559 10.1038/s41597-020-0501-z PMC7253458

[21] Kebede, E. A. *et al*. Assessing and addressing the global state of food production data scarcity. *Nat. Rev. Earth Environ*. 10.1038/s43017-024-00516-2 (2024).

[22] FAO. *Africa - Regional Overview of Food Security and Nutrition 2023*. 10.4060/cc8743en (FAO; AUC; United Nations Economic Commission for Africa (ECA); WFP, 2023).

[23] FEWS NET. FEWS NET Data Warehouse. https://help.fews.net/fdw (accessed on October 11, 2024) (2024).

[24] FEWS NET. FEWS NET Data Explorer. https://fdw.fews.net/data-explorer (accessed on October 11, 2024) (2024).

[25] UNSD. *Central Product Classification (CPC) Version 2.1*. https://unstats.un.org/unsd/classifications/econ/ (2015).

[26] Anderson, W. *et al*. Preseason maize and wheat yield forecasts for early warning of crop failure. *Nat. Commun.* **15**, 7262 (2024). 39179601 10.1038/s41467-024-51555-8 PMC11344146

[27] Lee, D. & Anderson, W. HarvestStat Africa. GitHub repository, https://github.com/HarvestStat/HarvestStat-Africa (accessed on February 20, 2025) (2024).

[28] Comenetz, J. Administrative Boundary Reorganization and the Mapping of Temporal Change. in (International Cartographic Association, Durban, South Africa, 2003).

[29] Fritz, S. *et al*. Mapping global cropland and field size. *Glob. Change Biol.* **21**, 1980–1992 (2015). 10.1111/gcb.12838 25640302

[30] Cerrani, I., *et al**Algorithm for the Disaggregation of Crop Area Statistics in the MARS Crop Yield Forecasting System*. (2023).

[31] Lee, D. *et al*. HarvestStat Africa - harmonized subnational crop statistics for Sub-Saharan Africa. *Dryad*10.5061/DRYAD.VQ83BK42W (2024). 10.1038/s41597-025-05001-z PMC12022251 40274821

[32] Ray, D. K., Ramankutty, N., Mueller, N. D., West, P. C. & Foley, J. A. Recent patterns of crop yield growth and stagnation. *Nat. Commun.* **3**, 1293 (2012). 23250423 10.1038/ncomms2296

[33] Iizumi, T. & Sakai, T. The global dataset of historical yields for major crops 1981–2016. *Sci. Data* **7**, 97 (2020). 32198349 10.1038/s41597-020-0433-7 PMC7083933

[34] Yu, Q. *et al*. A cultivated planet in 2010 – Part 2: The global gridded agricultural-production maps. *Earth Syst. Sci. Data* **12**, 3545–3572 (2020).

[35] Lee, D. *et al*. Contrasting performance of panel and time-series data models for subnational crop forecasting in Sub-Saharan Africa. *Agric. For. Meteorol.* **359**, 110213 (2024).

[36] Carletto, C. Better data, higher impact: improving agricultural data systems for societal change. *Eur. Rev. Agric. Econ.* **48**, 719–740 (2021).

[37] Olofsson, P. *et al*. Good practices for estimating area and assessing accuracy of land change. *Remote Sens. Environ.* **148**, 42–57 (2014).

[38] Peterson, S., Husak, G., Shukla, S. & McNally, A. Crop area change in the context of civil war in Tigray, Ethiopia. *Environ. Res. Food Syst.* **1**, 015003 (2024).

[39] Burke, M. & Lobell, D. B. Satellite-based assessment of yield variation and its determinants in smallholder African systems. *Proc. Natl. Acad. Sci.* **114**, 2189–2194 (2017). 28202728 10.1073/pnas.1616919114 PMC5338538

[40] Lobell, D. B. *et al*. Eyes in the Sky, Boots on the Ground: Assessing Satellite‐ and Ground‐Based Approaches to Crop Yield Measurement and Analysis. *Am. J. Agric. Econ.* **102**, 202–219 (2020).

[41] Gourlay, S., Kilic, T. & Lobell, D. B. A new spin on an old debate: Errors in farmer-reported production and their implications for inverse scale - Productivity relationship in Uganda. *J. Dev. Econ.* **141**, 102376 (2019).

