# Supplementary material for: HarvestStat Africa – Harmonized Subnational Crop Statistics for Sub-Saharan Africa
Source: Sci Data. 2025 Apr 24;12:690. doi: 10.1038/s41597-025-05001-z (PMC12022251; doi:10.1038/s41597-025-05001-z)
Supplement: Supplementary file 1 — Supplementary Information for “HarvestStat Africa – Harmonized Subnational Crop Statistics for Sub-Saharan Africa” [file 41597_2025_5001_MOESM1_ESM.pdf]

Supplementary Information for "HarvestStat Africa – Harmonized Subnational Crop Statistics for Sub-Saharan Africa"

Donghoon Lee[1,2,*], Weston Anderson[3,4,*], Xuan Chen[5], Frank Davenport[2], Shraddhanand Shukla[2], Ritvik Sahajpal[3], Michael Budde[6], James Rowland[6], Jim Verdin[7], Liangzhi You[5], Matthieu Ahouangbenon[8], Kyle Frankel Davis[8,9], Endalkachew Kebede[8], Steffen Ehrmann[10,11], Christina Justice[3], and Carsten Meyer[10,11,12]

[1] Department of Civil Engineering, University of Manitoba, Winnipeg, Manitoba, Canada
[2] Climate Hazards Center, Department of Geography, University of California, Santa Barbara, California, USA
[3] Department of Geographical Sciences, University of Maryland, College Park, Maryland, USA
[4] NASA Goddard Space Flight Center, Greenbelt, Maryland, USA
[5] International Food Policy Research Institute, Washington, DC, USA
[6] U.S. Geological Survey, Earth Resources Observation and Science Center, Sioux Falls, SD, USA
[7] U.S. Agency for International Development, Washington, DC, USA
[8] Department of Geography and Spatial Sciences, University of Delaware, Newark, DE 19716 USA
[9] Department of Plant and Soil Sciences, University of Delaware, Newark, DE 19716 USA
[10] German Centre for Integrative Biodiversity Research (iDiv) Halle-Jena-Leipzig, Leipzig, Germany
[11] Institute of Biology, Leipzig University, Leipzig, Germany
[12] Institute of Geosciences and Geography, Martin Luther University Halle-Wittenberg, Halle (Saale), Germany

Corresponding authors:

Donghoon Lee (Donghoon.Lee@umanitoba.ca) and Weston Anderson (Weston@umd.edu)

Summary:

This supplementary information includes two tables (Table S1 and Table S2).

Code and Data availability

Code and data supporting this study are publicly available. The processing scripts can be accessed via the HarvestStat Africa's GitHub repository[1], and the dataset (HarvestStat Africa v1.0) is hosted on Dryad[2]. Please refer to the repository and dataset for complete documentation and further details.

Disclaimer:

Any use of trade, firm, or product names is for descriptive purposes only and does not imply endorsement by the U.S. Government.

Table S1. List of source documents for crop statistics by country

Country	Source Documents
Angola	Official agricultural statistics, Angola, Ministry of Agriculture and Forestry
Benin	National Institute of Statistics and Economic Analysis (INSAE), Benin, benin.opendataforafrica.com
	National Institute of Statistics and Economic Analysis (INSAE), Benin, Annuaire statistique, 2010
	National Institute of Statistics and Economic Analysis (INSAE), Benin, Annuaire statistique, 2005-2007
	National Institute of Statistics and Economic Analysis (INSAE), Benin, Annuaire statistique, 2000-2004
	The Directorate of Agricultural Statistics (DSA), Ministère de l'Agriculture, de l'Elevage et de la Pêche du Bénin, Evolution de la Production, 1995-2020
	The Directorate of Agricultural Statistics (DSA), Ministère de l'Agriculture, de l'Elevage et de la Pêche du Bénin, Evolution de la Production, 2021-2022
	Institut National de la Statistique et de l'Analyse Économique, Benin, Compendium des Statistiques Agricoles et Alimentaires, 1970-1992
Burkina Faso	Institut National de la Statistique et de la Démographie (INSD), Burkina Faso, Annuaire statistique 2020 de la region du Nord
	Institut National de la Statistique et de la Démographie (INSD), Burkina Faso, Annuaire statistique 2012 de la region de l'Est
	Institut National de la Statistique et de la Démographie (INSD), Burkina Faso, Annuaire statistique 2012 de la region du Sahel
	Institut National de la Statistique et de la Démographie (INSD), Burkina Faso, Annuaire statistique 2020 de la region de l'Est
	Institut National de la Statistique et de la Démographie (INSD), Burkina Faso, Annuaire statistique 2020 de la region du Centre-Nord
	Ministry of Agriculture, Burkina Faso, FEWS AgroMaps
	Ministry of Agriculture, Burkina Faso, Official Spreadsheet 2017
	Ministry of Agriculture, Burkina Faso, Resultats de l'Enquete Permanente Agricole, Campagne 1995-1996
	Ministry of Agriculture, Burkina Faso, Resultats Definitifs de l'Enquete Permanente Agricole, Campagne 2015-2022
	Ministry of Agriculture, Burkina Faso, Resultats de l'Enquete Permanente Agricole, Campagne 2001-2002

Burundi	Institute of Statistics and Economic Studies, Burundi (ISTEEBU), Annuaire Statistique 2009
	Institute of Statistics and Economic Studies, Burundi (ISTEEBU), Annuaire Statistique 2014
	Ministere de l'Agriculture et de l'Elevage/DSIA, Burundi, Enquête Nationale Agricole du Burundi (ENAB) Rapport, 2011-2015
Cameroon	Ministere de l'agriculture, DEPA/CES, Cameroun, Official agricultural statistics
Chad	Ministry of Agriculture and Irrigation, Office Nationale du Developpement Rural (ONDR), Chad, Raport annuel, 1996-2020
Central African Republic	Directeur de la statistique agricole de la documentation et de l'informatique/Ministère du Développement Rural et de l'Agriculture, Central African Republic, Food and Agriculture Organization (FAO) and World Food Program, Joint Guidelines for Crop and Food Security Assessment Missions (CFSAM)
Democratic Republic of the Congo	Climate Change Strategy and Action Plan, Study and Intervention Group (CCSAP, SIG), Congo, The Democratic Republic of the, Crop and food security assessment mission
	SNSA, Congo, The Democratic Republic of the, Annuaire des Statistiques Agricoles (2006-2011)
Ethiopia	Central Statistics Agency, Ethiopia, Agricultural Sample Survey, 1995/96 - 2021/22, Meher
	Central Statistics Agency, Ethiopia, Agricultural Sample Survey, 2007/08 - 2021/22, Belg
Ghana	Ghana Statistical Services, Ghana, Statistics Research and Information Directorate (SRID) Spreadsheet, 2020
	Ghana Statistical Services, Ghana, Statistics Research and Information Directorate (SRID) Spreadsheet, 2022
	Ministry of Food and Agriculture (MOFA), Ghana, Sample Census of Agriculture, 1984
	Ministry of Food and Agriculture (MOFA), Ghana, CountryStat, December 2016
	L'Agence Nationale des Statistiques Agricoles et Alimentaires, Guinea, Annuaire Statistique 2014
Kenya	Agriculture and Food Authority (AFA), Kenya, AFA Year Book of Statistics, 2022
	Agriculture and Food Authority (AFA), Kenya, AFA Year Book of Statistics, 2024

	Agriculture and Food Authority (AFA), Kenya, AFA Year Book of Industrial Crops, 2016
	Ministry of Agricultural and Livestock Development, Kenya, FEWS AgroMaps, 2003
	Ministry of Agricultural and Livestock Development, Kenya, FEWS AgroMaps, 1996
	Ministry of Agricultural and Livestock Development, Kenya,KilimoSTAT, www.statistics.kilimo.go.ke, April 2024
	Ministry of Agricultural and Livestock Development, Kenya, National Maize Production 2014 - 2019
	Ministry of Agricultural and Livestock Development, Kenya, The Kenya Institute for Public Policy Research and Analysis (KIPPRA) Compendium 2003
	Ministry of Agricultural and Livestock Development, Kenya, Economic Review of Agriculture, 2013
Lesotho	Bureau of Statistics, Lesotho, Agricultural Production Survey, Crops, 2005-2022
	Bureau of Statistics, Lesotho, Agriculture Situation Report, 2010-11/2012-13
	Bureau of Statistics, Lesotho, Statistical Yearbook, 2010
	Bureau of Statistics, Lesotho, Statistical Yearbook, 2008
Liberia	Ministry of Agriculture, Liberia, FEWS Agman Archive
	Ministry of Agriculture, Liberia, Final calculation on production
	Ministry of Agriculture, Liberia, National Statistics Office
	Ministry of Agriculture, Liberia, Production Estimates of Major Crops and Animals, 2008
Madagascar	Ministry of Agriculture, Madagascar
	Ministry of Agriculture, Madagascar, FEWS AgroMaps, 2005
	Ministry of Agriculture, Madagascar, Annuaire des Statistiques Agricoles, 1993
	Ministry of Agriculture, Madagascar, Annuaire des Statistiques Agricoles, 2001-2004
	Ministry of Agriculture, Madagascar, Annuaire des Statistiques Agricoles, 1998-2001
	Ministry of Agriculture, Madagascar, Annuaire des Statistiques Agricoles, 1995, 1996, 1997
	Ministry of Agriculture, Madagascar, Annuaire des Statistiques Agricoles, 2005-2008

	Ministry of Agriculture, Madagascar, Official Spreadsheet, 2017
	Ministry of Agriculture, Madagascar, Annuaire des Statistiques Agricoles, 2007-2010
Malawi	Ministry of Agriculture, Irrigation and Water Development, Malawi, Ministry of Agriculture summary spreadsheet, 2002-2024
	National Statistical Office, Malawi, National Census of Agriculture and Livestock, 2006-2007
Mali	Direction Nationale de l'Agriculture/Direction Nationale de la Statistique et de l'Informatique, Mali, Analyse des donnees, 1982-1993
	Direction Nationale de l'Agriculture/Direction Nationale de la Statistique et de l'Informatique, Mali, CountryStat, 2023
	Direction Nationale de l'Agriculture/Direction Nationale de la Statistique et de l'Informatique, Mali, CountryStat, 2016
	Direction Nationale de l'Agriculture/Direction Nationale de la Statistique et de l'Informatique, Mali, Annuaire Statistique, 1998
	Direction Nationale de l'Agriculture/Direction Nationale de la Statistique et de l'Informatique, Mali, Annuaire Statistique, Gao, 1993
	Direction Nationale de l'Agriculture/Direction Nationale de la Statistique et de l'Informatique, Mali, FEWS AgroMaps, 2005
	Direction Nationale de l'Agriculture/Direction Nationale de la Statistique et de l'Informatique, Mali, Office Statistique des Communautes Europeenes (OSCE), Cereales et Elevage, 1989
Mauritania	Ministry of Rural Development, Mauritania, Enquête mauritanienne sur l'élevage et l'agriculture 2016-17
	Ministry of Rural Development, Mauritania, Division statistique agricole/DPSE/MDRE 2005-15 spreadsheet
	Ministry of Rural Development, Mauritania, Enquête mauritanienne sur l'élevage et l'agriculture 2010-14
	Ministry of Rural Development, Mauritania, Hodh ech Chargui en Chiffres, Edition 2014
	Ministry of Rural Development, Mauritania, Division statistique agricole/DPSE/MDRE, 1999-05 spreadsheet
	Ministry of Rural Development, Mauritania, Division statistique agricole/DPSE/MDRE 1989-93 spreadsheet
	Ministry of Rural Development, Mauritania, Synthese des resultats finaux de la saison normale de l'irrigue rizicole 2019
Mozambique	Instituto Nacional de Estatistica (INE), Mozambique, Indicadores Basicos de agricultura e alimentacao, 2015-2019

	Instituto Nacional de Estatistica (INE),Mozambique, Censo Agro-Pecuario 2009 - 2010: Resultados Definitivos - Mozambique
	Instituto Nacional de Estatistica (INE),Mozambique, Censo Agro-Pecuario 2009 - 2010: Resultados Preliminares - Mozambique
	Instituto Nacional de Estatistica (INE),Mozambique, Indicadores Basicos de Agricultura e Alimentacao, 2018-2022
	Ministerio da Agricultura e Seguranca Alimentar (MASA), Mozambique, Inquerito Agrario Integrado, 2020
	Ministerio da Agricultura e Seguranca Alimentar (MASA), Mozambique, Inquerito Agrario Integrado, 2002 - 2015
	Ministerio da Agricultura e Seguranca Alimentar (MASA), Mozambique, Analysis of maize production and yield in Mozambique (2000-2018): trends, challenges
Niger	Ministere de l'Agriculture, Niger, FEWS AgroMaps, 2006
	Ministere de l'Agriculture, Niger, Annuaire des Statistiques, 2009-2019
	Ministere de l'Agriculture, Niger, Rapport d'evaluation de la campagne agricole d'hivernage 2003 - 2007
	Ministere de l'Agriculture, Niger, Annuaire des Statistiques Regionales, 2017-2021
	Ministere de l'Agriculture, Niger, Rapport d'evaluation de la campagne agricole d'hivernage 2015
	Ministere de l'Agriculture, Niger, Rapport d'evaluation de la campagne agricole d'hivernage 2021
	Ministere de l'Agriculture, Niger, Rapport d'√©valuation de la campagne agricole d'hivernage 2023
Nigeria	National Agricultural Extension and Research Liaison Services (NAERLS), Nigeria, Crop Area and Output Forecast, 2014-2015
	National Agricultural Extension and Research Liaison Services (NAERLS), Nigeria, FEWS NET AgroMaps, 2006
	National Agricultural Extension and Research Liaison Services (NAERLS), Nigeria, FEWS NET AgroMaps, 2008
	National Agricultural Extension and Research Liaison Services (NAERLS), Nigeria, Report of the 2009 Agriculture Production Survey, 2009
	National Agricultural Extension and Research Liaison Services (NAERLS), Nigeria, Wet Season Agriculture Performance Survey 2006-2023
Rwanda	Ministere de l'Agriculture, de l'Elevage et des Forets, Service des Statistiques Agricoles, Rwanda, Official agricultural statistics

	Ministry of Agriculture and Animal Resources, Rwanda, Crop Assessment
	National Institute of Statistics Rwanda, Agricultural Survey
Senegal	Agence Nationale de la Statistique et de la Démographie (ANSD), Senegal, Situation Socio-Economique-Saint-Louis, 2004
	Agence Nationale de la Statistique et de la Démographie (ANSD), Senegal, Situation Socio-Economique-Saint-Louis, 2006
	Agence Nationale de la Statistique et de la Démographie (ANSD), Senegal, Situation Socio-Economique-Tambacounda, 2005-2008
	Agence Nationale de la Statistique et de la Démographie (ANSD), Senegal, Situation Socio-Economique-Dakar, 2004
	Agence Nationale de la Statistique et de la Démographie (ANSD), Senegal, Situation Socio-Economique-Dakar, 2013-2018
	Agence Nationale de la Statistique et de la Démographie (ANSD), Senegal, Situation Socio-Economique-Thies-2017-2018
	Agence Nationale de la Statistique et de la Démographie (ANSD), Senegal, Situation Socio-Economique-Thies-2012
	Agence Nationale de la Statistique et de la Démographie (ANSD), Senegal, Situation Socio-Economique-Saint-Louis 2010-2011
	Agence Nationale de la Statistique et de la Démographie (ANSD), Senegal, Situation Socio-Economique-Kolda-2010
	Agence Nationale de la Statistique et de la Démographie (ANSD), Senegal, Situation Socio-Economique-Kolda-2015
	Ministere de l'agriculture et du developpement rural, Direction de l'Analyse, de la Prévision et des Statistiques Agricoles (DAPSA), Senegal, http://senegalma.africadata.org/ (July 2016)
	Ministere de l'agriculture et du developpement rural, Direction de l'Analyse, de la Prévision et des Statistiques Agricoles (DAPSA), Senegal, FEWS AgroMaps, 2007
	Ministere de l'agriculture et du developpement rural, Direction de l'Analyse, de la Prévision et des Statistiques Agricoles (DAPSA), Senegal, Resultats previsionnels de la campagne agricole 2015
Sierra Leone	Ministry of Agriculture, Forestry and Food Security, Sierra Leone, Crop production, Sierra Leone
	Ministry of Agriculture, Forestry and Food Security, Sierra Leone, FEWS AgroMaps, Sierra Leone
Somalia	The Food Security and Nutrition Analysis Unit (FSNAU), Somalia, FSNAU Crop Assessment Spreadsheet 2023-24
South Africa	Crop Estimates Committee, South Africa, CEC Final Estimate, November 2022

	Crop Estimates Committee, South Africa, CEC Final Estimate, November 2021
	Crop Estimates Committee, South Africa, CEC Final Estimate, November 2020
	Crop Estimates Committee, South Africa, CEC Final Estimate, October 2019
	Crop Estimates Committee, South Africa, CEC Final Estimate, September 2018
	Crop Estimates Committee, South Africa, CEC Final Estimate, September 2017
	Crop Estimates Committee, South Africa, CEC Final Estimate, September 2016
	Crop Estimates Committee, South Africa, RSA Statistics Directorate database
South Sudan	FAO, WFP, Government of South Sudan (GoSS) , Crop Production Estimate , Crop and Food Security Assessment Mission (CFSAM), 2015 - 2022
Sudan	Federal Ministry of Agriculture and Forestry (FMoA&F), Sudan, Ministry of Agriculture spreadsheet
	Federal Ministry of Agriculture and Forestry (FMoA&F), Sudan, Time-Series of the Main Food and Oil Crops, 2015
Tanzania	Ministry of Agriculture, Food Security and Cooperatives, Tanzania, FEWS NET DALDO data, 2003
	Ministry of Agriculture, Food Security and Cooperatives (MAFC), Tanzania, Agriculture Basic Data 1998/99-2004/05 Booklet
	Ministry of Agriculture, Food Security and Cooperatives (MAFC), Tanzania, CountryStats.org
	Ministry of Agriculture, Livestock and Fisheries, Tanzania, Annual Agricultural Sample Survey Report, 2014/15
	National Bureau of Statistics, Tanzania, National Sample Census of Agriculture, 2002-2003
	National Bureau of Statistics, Tanzania, National Sample Census of Agriculture, 2007-2008
Togo	Direction des Statistiques Agricoles, de l'Informatique et de la Documentation, Togo, Evaluation de la Campagne Agricole, 1990-2013
	Direction des Statistiques Agricoles, de l'Informatique et de la Documentation, Togo, Country-Stat Togo, August 2016
	Direction des Statistiques Agricoles, de l'Informatique et de la Documentation, Togo, Annuaire Statistique de la Region Centrale, 2005-2012
	Institut National de la Statistique et des Etudes Economiques et Démographiques (INSEED), Togo, Annuaire Statistique de la Region Maritime, 2005-2012

	Institut National de la Statistique et des Etudes Economiques et Démographiques (INSEED), Togo, Annuaire Statistique de la Region de Kara, 2005-2014
	Institut National de la Statistique et des Etudes Economiques et Démographiques (INSEED), Togo, Annuaire Statistique Regional des Plateaux, 2019
	Institut National de la Statistique et des Etudes Economiques et Démographiques (INSEED), Togo, Annuaire Statistique National, 2020-2021
	Institut National de la Statistique et des Etudes Economiques et Démographiques (INSEED), Togo, Annuaire Statistique Regional de la Region de Kara, 2018-2019
Uganda	Bureau of Statistics, Uganda, Census of Agriculture, 2008-2009, Vol. IV
	Ministry of Agriculture, Animal Industry and Fisheries, Uganda, National Census of Agriculture and Livestock, 1990-1991, Vol. III
Zambia	Ministry of Agriculture and The Central Statistics Office, Zambia, National Census of Agriculture, 1990/1992, Part II, Vol. 2
	Ministry of Agriculture and The Central Statistics Office, Zambia, Crop Forecast Survey Results, 1976-2023
Zimbabwe	Ministry of Agriculture, AGRITEX, Zimbabwe, FEWS Office compilation, 2000
	Ministry of Agriculture (MoA), Zimbabwe, Second Round Crop and Livestock Assessment Report 2007/2008 - 2021/2022
	Ministry of Agriculture (MoA), Zimbabwe, Crop, Livestock and Fisheries Assessment Report (CLAFA) 2007/2008 - 2021/2022

Table S2. Crop types and data records for each country and season in HarvestStat Africa v1.0[2].

Country	Season (Record period)	Data records (Number of years)
Angola	Main (1997-2017)	Avocado (2), Banana (10), Beans (mixed) (16), Cabbage (3), Carrots (3), Cassava (17), Chili Pepper (3), Coffee (3), Cowpea (1), Garlic (4), Green Bean (1), Groundnuts (In Shell) (13), Lemon (2), Maize (21), Mango (1), Millet (9), Okras (3), Onions (3), Pineapple (3), Potato (12), Rice (8), Sorghum (6), Soybean (5), Sweet Potatoes (15), Tomato (2), Wheat (1)
Burkina Faso	Main (1984-2022)	Bambara groundnut (30), Cotton (23), Cowpea (30), Fonio (11), Groundnuts (In Shell) (32), Maize (32), Millet (33), Potato (3), Rice (34), Sesame Seed (26), Sorghum (33), Sorghum (Red) (13), Soybean (15), Sweet Potatoes (15), Yams (11)
	Annual (2015-2022)	Maize (4), Rice (7)
Burundi	Season A (1997-2016)	Banana (17), Beans (mixed) (15), Bush Bean (3), Cassava (17), Cowpea (1), Groundnuts (In Shell) (2), Maize (18), Millet (3), Pea (14), Pigeon Pea (0), Pole Bean (3), Potato (15), Rice (1), Sorghum (2), Soybean (1), Sunflower Seed (0), Sweet Potatoes (15), Taro (15), Wheat (2), Yams (5)
	Season B (1996-2014)	Banana (14), Beans (mixed) (14), Bush Bean (3), Cassava (17), Cowpea (2), Groundnuts (In Shell) (2), Maize (16), Millet (13), Pea (16), Pigeon Pea (2), Pole Bean (3), Potato (14), Rice (10), Sorghum (16), Soybean (2), Sunflower Seed (1), Sweet Potatoes (15), Taro (16), Wheat (10), Yams (8)
	Season C (1996-2014)	Banana (14), Beans (mixed) (13), Bush Bean (2), Cassava (17), Cowpea (0), Groundnuts (In Shell) (0), Maize (13), Millet (0), Pea (5), Pigeon Pea (1), Pole Bean (1), Potato (9), Rice (1), Sorghum (1), Soybean (0), Sunflower Seed (1), Sweet Potatoes (14), Taro (9), Wheat (1), Yams (0)
Benin	Main (1995-2021)	Bambara groundnut (23), Cowpea (26), Fonio (15), Geocarpa groundnut (18), Goussi (18), Groundnuts (In Shell) (26), Maize (26), Millet (19), Molokhia (5), Onions (7), Pigeon Pea (14), Potato (5), Sesame Seed (10), Sorghum (25), Soybean (18), Sugarcane (6), Sweet Potatoes (24), Taro (12), Watermelon (4), Yams (23)
	Annual (1995-2021)	Cabbage (4), Carrots (5), Cassava (26), Cucumber (4), Eggplant (11), Lettuce (8), Okras (20), Pineapple (10), Rice (22), Tomato (25)
DRC	Main (2005-2016)	Banana (10), Beans (mixed) (7), Cassava (10), Maize (10), Rice (10)
CAF	Main (2014-2016)	Cassava (3), Groundnuts (In Shell) (3), Maize (3), Rice (3), Sesame Seed (3)
Cameroon	Annual (1998-2008)	Bambara groundnut (3), Banana (8), Beans (mixed) (1), Cassava (8), Cowpea (4), Groundnuts (In Shell) (1), Maize (1), Melon (3), Millet (1), Okras (5), Onions (1), Pam Nut (7), Pineapple (5), Potato (1), Rice (2), Squash and Melon Seeds (1), Sweet Potatoes (1), Taro (10), Tomato (1), Watermelon (2), Yams (8)
	North 1st Season (1998-2008)	Beans (mixed) (9), Groundnuts (In Shell) (10), Maize (10), Millet (6), Potato (8), Rice (8), Sesame Seed (6), Soybean (8), Squash and Melon Seeds (8), Sweet Potatoes (9)
	North 2nd Season (1999-2008)	Beans (mixed) (7), Maize (6), Millet (5), Onions (10), Rice (6), Sweet Potatoes (6)
	1st Season (1998-2008)	Bambara groundnut (7), Beans (mixed) (6), Groundnuts (In Shell) (8), Maize (6), Melon (6), Millet (8), Potato (6), Rice (7), Sesame Seed (3), Soybean (6), Squash and Melon Seeds (7), Sweet Potatoes (7), Tomato (5), Watermelon (6)

	2nd Season (1998-2008)	Bambara groundnut (7), Beans (mixed) (6), Groundnuts (In Shell) (6), Maize (6), Melon (6), Millet (6), Potato (6), Rice (7), Soybean (6), Squash and Melon Seeds (5), Sweet Potatoes (7), Tomato (5), Watermelon (6)
Ethiopia	Meher (1998-2016)	Avocado (5), Banana (7), Barley (12), Beans (White) (9), Beet (3), Cabbage (4), Carrots (1), Chick Peas (8), Chili Pepper (3), Coffee (7), Ethiopian Cabbage (4), Fava Bean (13), Fenugreek (7), Field Peas (12), Garlic (4), Green Peppers (3), Groundnuts (In Shell) (5), Hops (7), Lemon (2), Lentils (9), Linseed (9), Maize (14), Mango (5), Millet (8), Mung bean (3), Neug (10), Oats (5), Onions (3), Orange (4), Papaya (5), Pineapple (1), Potato (6), Rape (6), Rice (1), Sesame Seed (6), Sorghum (12), Soybean (3), Sugarcane (6), Sunflower Seed (3), Sweet Potatoes (7), Taro (4), Teff (15), Tomato (1), Wheat (13), Yams (1)
Ghana	Annual (1997-2018)	Banana (22), Cassava (22), Taro (21)
	Main (1984-2022)	Banana (3), Cassava (4), Cowpea (18), Groundnuts (In Shell) (17), Maize (26), Millet (26), Rice (25), Sorghum (22), Soybean (17), Sweet Potatoes (8), Taro (4), Yams (23)
	Main (2010-2015)	Cassava (6), Groundnuts (In Shell) (6), Maize (6), Rice (6)
Kenya	Annual (1965-2022)	Bambara groundnut (6), Banana (5), Barley (2), Beans (mixed) (35), Cabbage (5), Canola Seed (4), Carrots (2), Cashew (unshelled) (8), Cassava (8), Coffee (10), Coriander (4), Cotton (14), Cowpea (8), Groundnuts (In Shell) (7), Jute (7), Kale (6), Macadamia (6), Maize (34), Millet (22), Mung bean (7), Onions (3), Pea (4), Pigeon Pea (1), Pineapple (4), Potato (17), Pyrethrum (14), Rice (13), Sesame Seed (22), Sorghum (11), Sugarcane (5), Sunflower Seed (14), Sweet Potatoes (8), Taro (1), Tea (12), Tobacco (16), Tomato (5), Watermelon (6), Wheat (27), Yams (1)
	Long (1991-2024)	Beans (mixed) (7), Maize (19), Sorghum (1)
	Short (1991-2024)	Beans (mixed) (6), Maize (12), Sorghum (2)
Liberia	Main (1995-2015)	Cassava (7), Rice (10)
Lesotho	Summer (1981-2022)	Beans (mixed) (36), Maize (39), Oats (0), Pea (30), Sorghum (38), Wheat (34)
	Winter (2006-2022)	Beans (mixed) (0), Maize (1), Oats (0), Pea (9), Sorghum (0), Wheat (7)
Madagascar	Annual (1987-2019)	Bambara groundnut (1), Banana (1), Barley (1), Beans (mixed) (21), Beet (1), Carrots (1), Cassava (28), Chili Pepper (1), Coffee (19), Cotton (1), Cowpea (1), Cucumber (1), Eggplant (1), Garlic (1), Ginger (1), Green Pea (1), Groundnuts (In Shell) (18), Jute (1), Lentils (1), Lettuce (1), Maize (28), Millet (1), Onions (1), Pepper (1), Pigeon Pea (18), Pineapple (1), Potato (11), Rice (30), Soybean (1), Squash (1), Sugarcane (21), Sweet Potatoes (23), Taro (1), Tobacco (1), Tomato (1), Wheat (1), Yams (1)
Mali	Main (1974-2022)	Bambara groundnut (32), Barley (2), Beans (mixed) (4), Cotton (35), Cowpea (28), Fonio (35), Groundnuts (In Shell) (34), Maize (36), Millet (37), Rice (36), Sesame Seed (16), Sorghum (37), Soybean (8), Sugarcane (26), Sweet Potatoes (4), Tomato (1), Wheat (11), Yams (6)
Mauritania	Annual (1989-2019)	Cowpea (4), Groundnuts (In Shell) (1), Maize (4), Millet (4), Rice (19), Sorghum (5)
	Bas-fond (1999-2016)	Cowpea (1), Maize (9), Rice (1), Sorghum (12), Wheat (3)

	Dam retention (1999-2016)	Cowpea (1), Maize (5), Rice (0), Sorghum (4), Wheat (2)
	Main (1999-2016)	Cowpea (1), Maize (7), Millet (12), Sorghum (14)
	Walo (1999-2016)	Cowpea (1), Maize (9), Sorghum (13)
	Decrue controlee (2000-2016)	Maize (5), Sorghum (7)
	Hot off-season (2005-2016)	Rice (7)
	Cold off-season (2010-2016)	Wheat (3)
Malawi	Main (1983-2020)	Bambara groundnut (15), Banana (3), Bean (Hyacinth) (13), Beans (mixed) (12), Cabbage (2), Cassava (31), Chick Peas (8), Chili Pepper (9), Coffee (8), Cotton (27), Cowpea (15), Field Peas (10), Garlic (1), Groundnuts (In Shell) (30), Maize (34), Millet (23), Onions (2), Paprika (9), Pigeon Pea (14), Potato (14), Rice (22), Sesame Seed (11), Sorghum (25), Soybean (14), Sunflower Seed (11), Sweet Potatoes (17), Tobacco (14), Tomato (2), Velvet Bean (12)
	Annual (2018-2023)	Beans (mixed) (3), Cassava (3), Groundnuts (In Shell) (3), Maize (3), Rice (3), Soybean (3)
	Winter (2006-2020)	Beans (mixed) (12), Cabbage (2), Cowpea (11), Field Peas (10), Garlic (1), Onions (2), Paprika (4), Pigeon Pea (1), Potato (11), Sweet Potatoes (13), Tomato (2)
Mozambique	Main (1999-2022)	Bambara groundnut (20), Beans (Rosecoco) (12), Beans (mixed) (15), Chili Pepper (2), Cowpea (20), Ginger (1), Green Bean (6), Groundnuts (In Shell) (13), Maize (31), Millet (19), Mung bean (3), Paprika (2), Pepper (2), Pigeon Pea (19), Sesame Seed (12), Sorghum (25), Soybean (2), Sugarcane (1), Sunflower Seed (8), Sweet Potatoes (5), Tobacco (12), Virginia Peanut (20), Wheat (1)
	Annual (1999-2022)	Cashew (unshelled) (7), Cassava (26), Jute (4), Macadamia (2), Sugarcane (3), Tea (3)
	Cotton season (1999-2020)	Cotton (16)
	Rice season (1999-2022)	Banana (2), Rice (26)
Niger	Dry (2011-2022)	Bean (Hyacinth) (2), Cabbage (7), Capsicum Chinense (6), Carrots (6), Cassava (5), Celery (2), Chili Pepper (4), Coriander (2), Cowpea (5), Cucumber (1), Eggplant (3), Garlic (3), Groundnuts (In Shell) (2), Lettuce (7), Maize (6), Melon (3), Okras (4), Onions (7), Pea (2), Potato (6), Rape (3), Rice (3), Sorghum (3), Sorrel (1), Squash (6), Sugarcane (5), Sweet Potatoes (5), Tobacco (2), Tomato (7), Watermelon (3), Wheat (3)
	Main (1980-2022)	Bambara groundnut (10), Cabbage (1), Capsicum Chinense (1), Cassava (1), Chili Pepper (1), Cotton (2), Cowpea (32), Cucumber (1), Fonio (8), Groundnuts (In Shell) (22), Lettuce (1), Maize (12), Millet (36), Okras (8), Onions (5), Potato (0), Rice (11), Sesame Seed (14), Sorghum (36), Sorrel (9), Squash (1), Sugarcane (1), Sweet Potatoes (1), Tomato (2)

Nigeria	Wet (1999-2023)	Banana (2), Cassava (2), Cotton (16), Cowpea (23), Ginger (9), Groundnuts (In Shell) (21), Maize (24), Melon (8), Millet (19), Okras (10), Onions (11), Rice (24), Sesame Seed (12), Sorghum (19), Soybean (17), Sweet Potatoes (10), Tomato (12), Wheat (7)
	Annual (1999-2023)	Cassava (22), Taro (18), Yams (23)
Rwanda	Season A (2008-2017)	Avocado (1), Banana (4), Beans (mixed) (1), Beet (1), Bush Bean (2), Cabbage (1), Carrots (1), Cassava (3), Celery (1), Cereal Crops (0), Eggplant (1), Green Bean (1), Green Pea (1), Groundnuts (In Shell) (3), Maize (4), Okras (1), Pea (3), Pole Bean (3), Potato (4), Rice (2), Sorghum (2), Soybean (3), Squash (1), Sugarcane (1), Sunflower Seed (1), Sweet Potatoes (4), Taro (1), Tomato (1), Wheat (2), Yams (3)
	Season B (2008-2017)	Avocado (1), Banana (4), Beans (mixed) (1), Beet (1), Bush Bean (2), Cabbage (1), Carrots (1), Cassava (4), Celery (1), Cereal Crops (1), Eggplant (1), Green Bean (1), Green Pea (1), Groundnuts (In Shell) (3), Maize (4), Okras (1), Pea (3), Pole Bean (2), Potato (3), Rice (3), Sorghum (3), Soybean (3), Squash (1), Sugarcane (1), Sunflower Seed (1), Sweet Potatoes (4), Taro (1), Tomato (1), Wheat (2), Yams (3)
	Season C (2013-2013)	Bush Bean (0), Pea (1), Pole Bean (0), Potato (1), Soybean (1)
Sudan	Annual (1976-2024)	Cotton (3), Cotton (Acala) (6), Sorghum (22), Wheat (19)
	Main (1975-2023)	Cotton (5), Cotton (American) (11), Groundnuts (In Shell) (28), Millet (55), Pigeon Pea (1), Sesame Seed (42), Sorghum (59), Sunflower Seed (14), Wheat (8)
Sierra Leone	Main (1986-2016)	Banana (0), Cashew (unshelled) (0), Cassava (2), Groundnuts (In Shell) (2), Maize (2), Millet (0), Okras (2), Potato (0), Rice (2), Sesame Seed (2), Sorghum (2), Sweet Potatoes (2)
Senegal	Main (1960-2015)	Cassava (7), Cowpea (35), Fonio (6), Groundnuts (In Shell) (48), Maize (35), Millet (46), Rice (33), Sesame Seed (4), Sorghum (25), Sweet Potatoes (1)
	Main-off (2000-2011)	Groundnuts (In Shell) (3), Maize (6), Rice (8)
Somalia	Deyr (1996-2023)	Cowpea (8), Groundnuts (In Shell) (7), Maize (21), Onions (6), Pepper (9), Rice (5), Sesame Seed (10), Sorghum (17), Tomato (4), Watermelon (4)
	Gu (1995-2021)	Cowpea (9), Groundnuts (In Shell) (5), Maize (23), Onions (8), Pepper (9), Rice (11), Sesame Seed (8), Sorghum (18), Tomato (5), Watermelon (3)
	Deyr-off (2004-2021)	Cowpea (6), Maize (4), Sesame Seed (3), Sorghum (1)
	Gu-off (2005-2019)	Cowpea (4), Maize (5), Sesame Seed (5), Sorghum (2)
South Sudan	Main (1975-2013)	Cereal Crops (2), Cotton (American) (4), Groundnuts (In Shell) (23), Millet (30), Sesame Seed (31), Sorghum (43), Sunflower Seed (4)
	Annual (1997-2010)	Cotton (Acala) (10), Sorghum (11)
Chad	Main (1983-2017)	Bambara groundnut (9), Cassava (14), Cowpea (17), Fonio (4), Groundnuts (In Shell) (24), Maize (25), Millet (31), Rice (21), Sesame Seed (21), Sorghum (29), Sweet Potatoes (6), Taro (6), Wheat (28)
	Cold-off (1983-2017)	Sorghum (19)

Togo	Main (1995-2015)	Beans (mixed) (5), Cassava (5), Cotton (4), Cowpea (5), Groundnuts (In Shell) (5), Maize (19), Millet (9), Sorghum (16), Soybean (1), Sweet Potatoes (0), Yams (4)
	Annual (2005-2015)	Rice (4)
Tanzania	Long (2003-2015)	Bambara groundnut (2), Barley (1), Beans (mixed) (1), Cassava (1), Chick Peas (1), Cowpea (1), Field Peas (1), Groundnuts (In Shell) (2), Maize (2), Millet (2), Mung bean (1), Pigeon Pea (1), Potato (1), Rice (2), Sesame Seed (1), Sorghum (2), Soybean (1), Sunflower Seed (1), Sweet Potatoes (1), Taro (1), Wheat (1), Yams (1)
	Annual (1989-2015)	Bambara groundnut (3), Banana (15), Barley (9), Beans (mixed) (12), Cassava (18), Chick Peas (2), Cowpea (4), Field Peas (6), Groundnuts (In Shell) (12), Maize (21), Millet (12), Mung bean (3), Pea (2), Pigeon Pea (1), Potato (10), Rice (19), Sesame Seed (9), Sorghum (18), Soybean (3), Sugarcane (0), Sunflower Seed (8), Sweet Potatoes (13), Taro (1), Wheat (11), Yams (1)
	Short (2003-2015)	Bambara groundnut (1), Barley (1), Beans (mixed) (1), Cassava (1), Chick Peas (1), Cowpea (1), Field Peas (1), Groundnuts (In Shell) (1), Maize (2), Millet (1), Mung bean (1), Pigeon Pea (1), Potato (1), Rice (2), Sesame Seed (1), Sorghum (1), Soybean (1), Sunflower Seed (1), Sweet Potatoes (1), Taro (1), Wheat (1), Yams (1)
	Long/Dry (2003-2003)	Cassava (1), Chick Peas (1), Maize (1), Mung bean (1), Soybean (1), Taro (1)
Uganda	First (2009-2009)	Banana (0), Beans (mixed) (0), Cassava (0), Cowpea (0), Field Peas (0), Groundnuts (In Shell) (0), Maize (1), Millet (0), Potato (0), Rice (0), Sesame Seed (0), Sorghum (0), Soybean (0), Sweet Potatoes (0)
	Second (2008-2008)	Banana (0), Beans (mixed) (1), Cassava (1), Cowpea (0), Field Peas (0), Groundnuts (In Shell) (1), Maize (1), Millet (0), Potato (0), Rice (0), Sesame Seed (0), Sorghum (0), Soybean (0), Sweet Potatoes (0)
	Annual (2008-2009)	Pigeon Pea (1)
South Africa	Winter (1979-2022)	Barley (11), Canola Seed (17), Wheat (44)
	Summer (1981-2022)	Beans (mixed) (29), Groundnuts (In Shell) (36), Maize (35), Maize (Yellow) (35), Sorghum (25), Soybean (31), Sunflower Seed (26)
Zambia	Annual (1980-2017)	Bambara groundnut (8), Barley (2), Beans (mixed) (19), Cassava (0), Coffee (1), Cottonseed (11), Cowpea (9), Maize (33), Millet (16), Pineapple (1), Potato (6), Rice (14), Sorghum (18), Soybean (17), Sugarcane (1), Sunflower Seed (15), Sweet Potatoes (16), Velvet Bean (1), Wheat (6)
Zimbabwe	Main (1981-2023)	Bambara groundnut (1), Beans (Rosecoco) (11), Cassava (0), Cowpea (6), Groundnuts (In Shell) (31), Maize (52), Millet (41), Rape (7), Rice (3), Sesame Seed (4), Sorghum (34), Soybean (21), Sunflower Seed (24), Sweet Potatoes (8)

References

1. Lee, D. & Anderson, W. HarvestStat Africa. GitHub repository, https://github.com/HarvestStat/HarvestStat-Africa (accessed on February 20, 2025) (2024).
2. Lee, D. *et al.* HarvestStat Africa - harmonized subnational crop statistics for Sub-Saharan Africa. Dryad https://doi.org/10.5061/DRYAD.VQ83BK42W (2024).
